# Challenges in classifying human chromosomal heteromorphisms using banding cytogenetics: From controversial guidelines to the need for a universal scoring system

**DOI:** 10.1038/s41439-024-00295-8

**Published:** 2024-10-24

**Authors:** Sílvia Pires, Paula Jorge, Thomas Liehr, Natália Oliva-Teles

**Affiliations:** 1 Centro Hospitalar Universitário de Santo António, Unidade Local de Saúde de Santo António, Porto, Portugal; 2 Unit for Multidisciplinary Research in Biomedicine, Institute of Biomedical Sciences Abel Salazar, University of Porto, Porto, Portugal; 3 Department of Microscopy, Institute of Biomedical Sciences Abel Salazar, University of Porto, Porto, Portugal; 4 Laboratory for Integrative and Translational Research in Population Health, Porto, Portugal; 5 Jena University Hospital, Friedrich Schiller University, Institute of Human Genetics, Jena, Germany; 6 Center of Bioethics, University of Porto, Porto, Portugal

**Keywords:** Cytogenetics, Structural variation, Genomic analysis

## Abstract

Chromosomal heteromorphisms (CHs) are morphological variations predominantly found in constitutive heterochromatic regions of the genome, primarily composed of tandemly repetitive sequences of satellite DNA. Although not completely devoid of genes, these regions are typically not transcribed into proteins and lack obvious phenotypic impact. Nonetheless, their clinical importance is increasingly under scrutiny, with several studies aiming to assess their influence on human diseases and susceptibilities, especially as they are seemingly part of the long noncoding RNAs in certain tissues. This article summarizes the classification methods of human heterochromatic CHs documented in the literature over the last two decades. Multiple scoring systems have been identified, and previous approaches for CH assessment and reporting in genetic diagnosis have shown inconsistencies. Owing to the current heterogeneity in the classification of CHs, data analysis may be biased, impacting the quality of clinical reports and human genetic research. This review highlights the need for a universal scoring system, which is essential for scientific reproducibility and the accurate identification and clinical evaluation of human CHs.

## Standardizing chromosomal heteromorphisms for better genetic insights

In 2004, the Human Genome Project revealed the complete human DNA sequence, showing that most of our genome consists of noncoding and repetitive DNA sequences. Researchers reviewed various classification systems for chromosomal heteromorphisms to address this knowledge gap. They conducted a literature review using PubMed/MEDLINE, focusing on studies from 2000 to 2024 that described or used classification systems for CHs. This review included 18 studies and identified three main classification methods: Linear Measurement, Twice the Size of the Homologous, and 16p Size Comparison. The results showed inconsistencies in evaluating CHs due to different methodologies and lack of standard sizes for heterochromatic regions. Researchers concluded that standardizing CH classification is crucial for accurate genetic diagnosis and scientific reproducibility. Future research should focus on developing clear guidelines and advanced detection technologies to better understand CHs and their clinical implications. "This summary was initially drafted using artificial intelligence, then revised and fact-checked by the author."

## Introduction

In 2004, the Human Genome Project provided comprehensive information on the human DNA sequence and redefined paradigms^1^. The majority of our genome consists of noncoding and/or repetitive DNA sequences, which underlie the heteromorphisms and polymorphisms identified at the chromosomal and molecular levels^2,3^. These sequences can be organized tandemly or as single repeat units spread throughout the genome and can be categorized according to the size and motif of the repeats, such as microsatellite, minisatellite and satellite DNAs^3,4^. Satellite DNA families (I, II, III, α, ß and γ) cover approximately 10% of the human genome and are often organized into higher-order repeat units (HORs), particularly in the centromeric regions of eukaryotic chromosomes^5^. HOR structures influence regulatory, replication, and gene expression processes, and the specific composition of their monomers plays a crucial role in defining a unique centromere identity, which also contributes to population diversity^6^.

In human karyotypes, variant heterochromatic portions of repetitive DNA have been recognized and documented for decades^7^. These regions consist of highly repetitive sequences, composed of micro- and minisatellites; α-, β- and other satellite DNAs; and transposable elements, which vary in extent from five to a few hundred base pairs^8^. Microscopically visible variations in these sections are commonly observed during standard cytogenetic examinations. Defined as chromosomal heteromorphisms (CHs), these alterations include differences in size, morphology and/or banding patterns of the constitutive heterochromatin segments. CHs are usually inherited and are typically located in 1) the centromeric regions of any of the human chromosomes; 2) the short arms, satellites, or stalks of the acrocentric chromosomes; or 3) the pericentromeric region of chromosomes 1, 9, and 16 and the long arm of Y chromosome^9^. Although not devoid of genes, these segments are generally not actively transcribed or expressed and are regarded as genetically inert^10,11^. CHs present in 2–5% of the general population are considered mitotically stable normal variants, and clinical practice cytogenetic guidelines recommend not reporting them^12–14^.

Despite having been widely accepted as benign, several studies concerning CHs have yielded conflicting results regarding their impact on human health^15^. Numerous authors have proposed an association between the presence of heteromorphic variants and clinical phenotypes such as infertility, neurodegenerative disorders or cancer predisposition^4,7,16–18^. The etiological mechanisms behind these correlations remain elusive; however, scientific studies suggest a potential impact of CHs on genome stability and modulation. The prevailing hypotheses point toward a likely effect of CHs on meiotic chromosome pairing and segregation and genome regulation, by either modulating or inhibiting the expression of specific genes through position-effect variegation^8,11,19,20^. Previous studies have revealed that satellite DNAs are transcribed as long noncoding RNAs (lncRNAs) in advanced cancer, under cellular stress and during embryogenesis (contributing to cellular specification and differentiation), highlighting the influence of repetitive DNA in biological processes^21–23^.

In addition to the relevance of clinical and genetic assessments of heteromorphic variants, CHs offer a valuable resource for evolutionary and population research because they are inherited in a Mendelian pattern and have low mutation rates. Therefore, CHs can serve as markers and can be used to identify and determine genetic linkages^4^.

Fully understanding CHs is crucial for both basic and clinical research, yet heterochromatic regions cannot be easily sequenced and aligned. As a result, CHs remain poorly documented in human genome browsers and are still exclusively identified using (molecular) cytogenetic methods^16,24^. Although these variations have been identified for many years, there is no consensus within the cytogenetic scientific community regarding the “normal” sizes of CHs or the adoption of a unique classification system for their evaluation^9,15^. Since the Paris Cytogenetic Conference in 1971, in which the first CH assessment scale was established, several classification methods have been proposed^25–28^. However, cytogenetic guidelines do not establish a standard scoring system, and approaches for evaluating CHs and their clinical and functional roles are inconsistent^9,15^.

This article provides a short review of the various classification systems for human heterochromatic CHs reported in the literature over the last two decades. Although “euchromatic variants” are also the result of large euchromatic copy number variations (CNVs), these variants are not considered in this review^28^. The aim is to identify, describe and critically evaluate different methodological approaches to improve and standardize the classification of human CHs in the future.

## Materials and Methods

### Searching strategy

The literature review was performed using the PubMed/MEDLINE electronic database. The search was restricted to articles written in the English language and published between 1 January 2000 and 30 April 2024. The key concepts used for the search in the title and/or abstract of all papers were “chromosome/chromosomal heteromorphisms”, “chromosome/chromosomal polymorphisms”, “heterochromatin variants/polymorphisms”, “size polymorphisms” and “heteromorphisms classification”. An additional “human species” filter was applied. The Boolean operators used were “AND”, “OR” and “NOT”.

### Study selection: inclusion and exclusion criteria

With respect to the inclusion criteria, we selected only studies that described or used, as a methodological tool, classification systems for human heterochromatic CHs. Additionally, data suggesting a scoring system from a book by Thomas Liehr in 2014 were included^28^. All the articles were screened on the basis of their titles and abstracts to identify those meeting the inclusion criteria. The full text of each selected paper was subsequently assessed. Papers that evaluated human CHs but omitted the classification system were excluded.

### Data extraction

The following information was extracted from the selected articles: (1) study identification (author and year); (2) article type; (3) scoring system; (4) banding technique; and (5) chromosomes assessed.

## Results

### Review of the literature

A total of 31 articles and their own source^28^ were identified and screened for inclusion. Fourteen papers were excluded because of the omission of a classification system. Among the 18 selected publications, several scoring systems with numerous variations each were identified. To clarify and simplify the scientific discussion, we grouped scoring systems into three main types on the basis of the use of different methodologies, rating levels and specific chromosome/chromosomal sections to which each system could be applied (1 - CHs at the centromeres of all chromosomes; 2 - CHs at the short arms of the acrocentric chromosomes; 3 - CH susceptible regions of chromosomes 1, 9, 16 and Y; and 4 - specific pericentric inversions). Twelve papers described/used the “twice the size of the homologous” method (TSH), three described/used the “16p size comparison” model (16p SC) and two described/used the “linear measurement” method (LM). One of the articles does not fit into any of these three main groups and therefore will not be categorized^29^. Information on the articles used in this review is provided in Table 1.

**Table 1:** List of reviewed articles that met the stipulated inclusion criteria, ordered by year of publication.

Data Source	Article Type	Classification System	Banding Technique	Assessed Chromosomes
Yasseen et al.^30^	Original Research	LM	C	1 9 16
Yasseen et al.^31^	Original Research	LM	C	Y
Madon et al.^34^	Original Research	TSH	G, Q	9 13 14 15 21 22 Y
Sahin et al.^32^	Original Research	TSH + G group SC*	G, C, NOR	1 9 13 14 15 16 21 22 Y
Minocherhomji et al.^38^	Original Research	TSH	G, C, DAPI	1 9 13 14 15 16 21 22 Y
Martínez et al. 2012	Original Research	F group SC*	G	Y
Akbas et al. 2012	Original Research	TSH + G group SC*	G, C, NOR	1 9 13 14 15 16 21 22 Y
Guo et al.^35^	Original Research	TSH	G	1 9 13 14 15 16 21 22 Y
Mierla et al.^39^	Original Research	TSH	G	1 9 13 14 15 16 21 22 Y
Liehr 2014	Book	16p SC + 18p SC**	G	1 9 13 14 15 16 21 22 Y
Liehr 2016	Review	16p SC + 18p SC**	G	1 9 13 14 15 16 21 22 Y
Zhu et al.^37^	Original Research	TSH + G group SC*	G, C, NOR	1 9 13 14 15 16 21 22 Y
Rawal et al.^33^	Original Research	TSH	G	1 9 13 14 15 21 22 Y
Karaca et al.^15^	Original Research	16p SC + TSH***	G	1 9 13 14 15 16 21 22 Y
Li et al.^19^	Original Research	TSH	G, C, NOR	1 9 13 14 15 16 21 22
Chakraborty et al.^40^	Original Research	TSH	G, NOR	1 9 13 14 15 16 21 22 Y
Rodriguez et al.^41^	Original Research	TSH	G	1 9 13 14 15 16 21 22 Y
Mottola et al.^18^	Original Research	TSH	G	9

*LM* linear measurement method, *TSH* twice the size of the homologous method, *16p SC* 16p size comparison model.

*F or G group size comparison models are exclusively used for the Y chromosome.

**The 18p size comparison model is exclusively used for the acrocentric chromosomes.

***TSH method is exclusively used for the acrocentric chromosomes.

### Linear measurement method

Introduced in 1978 by Balicek et al., the LM technique determines the precise length of the constitutive heterochromatin regions on chromosomes 1, 9, 16, and Y^27^. On the basis of a five-step assessment scale of C-band lengths proposed at the Paris Conference, the authors ascribed exact numerical limits to each category. The determination of the C-band length is conducted directly from an image using linear measurements, with the absolute dimensions expressed in micrometers. Table 2 presents the proposed limits for each chromosome. Originally used for paternity testing, the LM methodology was documented in two of the selected articles, where it was applied to evaluate the influence of CHs on male infertility^27,30,31^.

**Table 2:** Five-step evaluation scale of C-band length limits (in units of 10^−^^7^ m) for chromosomes 1, 9, 16 and Y as proposed by Balicek et al.

Chromosome	Very small	Small	Intermediate	Large	Very Large
1	−7.2	7.3–10.0	10.1–15.4	15.5–18.4	18.5-
9	−6.2	6.3–8.3	8.4–13.6	13.7–15.5	15.6-
16	−3.8	3.9–5.3	5.4–8.9	9.0–10.7	10.8-
Y	−8.0	8.1–8.6	8.7–12.2	12.3–12.9	13.0-

### Twice the size of the homologous method

This methodology is based on direct comparison of the size of the heterochromatic regions between homologous chromosomes of the same metaphase spread. On autosomes, variants must be at least twice or half the size of the corresponding region on its homolog and are reported as “h + ” or “h-”, respectively. The prominent satellites and stalks of the acrocentric chromosomes are referred to as “ps + ” and “pstk + ”, respectively. The length of the Y chromosome is compared with that of G-group chromosomes and reported as “Yqh + /-”, if larger or smaller, respectively. Pericentric inversions are described as “inv”. This evaluation system can be used directly in metaphases with G-banding or after selective banding, such as the C and NOR (nucleolar organizing region using silver stain) techniques. In 12 of the analyzed papers, this methodology was generally used as a tool to assess the clinical impact of CHs on human fertility^18,19,32–41^.

### 16p size comparison model

**Fig. 1:**
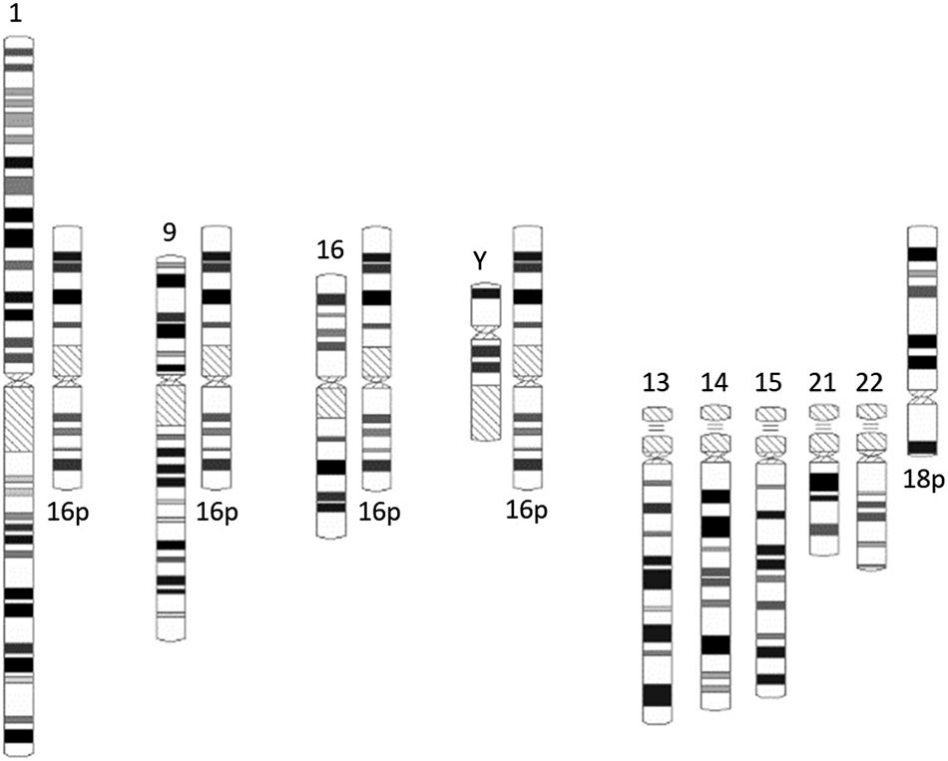
The 16p size comparison model adapted by Liehr (2016). In this CH classification, the heteromorphic regions of chromosomes 1, 9, 16 and Y are compared to the short arm of chromosome 16, and the short arms of all the acrocentric chromosomes are compared to the short arm of chromosome 18. For the purpose of demonstration, the ideograms of chromosomes 16 and 18 are shown upside down next to each heteromorphic region as references.

Originally developed in 1978 by Verma et al. for C banding, size heteromorphisms were classified into levels using 16p as a reference standard^26^. This methodology evaluates the “qh” regions on chromosomes 1, 9, 16 and Y by comparing their size with the short arm of chromosome 16 at the same G- or C-banded metaphase spread. Subsequently, heterochromatic regions can be scored as levels 1 to 5 on the basis of their length relative to the length of 16p (Table 3) or can be classified as “qh + ” variants if they are greater than 16p and “qh-” variants if they are less than half of 16p. TSH methodology is often used to assess the sizes of the short arms, stalks and satellites of acrocentric chromosomes. In 1977, Verma et al. suggested 18p as a reference standard for evaluating the sizes of the short arms of acrocentric chromosomes (“p + /−” variants) (Fig. 1)^42^. In 2016, Liehr suggested the diameter of a chromatid from the same metaphase as a size reference to evaluate the centromeric regions^9^. The 16p SC model was documented in three of the selected sources, where it was applied as a research and diagnostic tool^9,15,28^.

**Table 3:** Heterochromatin region size levels, adapted from Verma et al. and detailed by Karaca et al.^15^.

Level	16p size ratio	Size classification
1	0.5 < x 16p	Very small
2	≥ 0.5–1 x 16p	Small
3	> 1–1.5 x 16p	Intermediate
4	> 1.5–2 x 16p	Large
5	> 2 x 16p	Very large

## Discussion

Constitutive heterochromatin comprises approximately 45% of the human genome, with chromosomal heteromorphic regions constituting at least 10% of the latter^10,17^. Each individual not only possesses a distinctive DNA primary sequence but also carries a unique combination of these heterochromatin variants. In the human karyotype, the major CHs are located at 1q12, 9q12, 13pter-q11, 14pter-q11.1, 15pter-q11.1, 16q11.2, 19p12-q12, 21pter-q11.1, 22pter-q11.1 and Yq12^43^. The reported incidence of each subgroup of CHs may be subject to variations on the basis of the ethnic origin of the population and the cytogenetic methodologies and observation criteria applied^28^. At the Paris Cytogenetic Conference in 1971, a system for evaluating human CHs was proposed for the first time. The lengths of the heterochromatin regions were visually categorized into five groups on the basis of their size in C-banding and intensity levels in Q-banding: very small, small, intermediate, large, and very large^44^. Several CH classification methods have been presented, and cytogenetic variants have been classified by position, size and staining intensity, using diverse selective banding techniques, such as CBG (C-bands by barium hydroxide using Giemsa), QFQ (Q-bands by fluorescence using quinacrine), NOR and DAPI (distamycin A/DAPI banding)^45^. CHs described in the early stages have been summarized in previous Human Cytogenetic/Genomic Nomenclature Conferences. In recent decades, CHs have also started to be classified using standard G-banding and are documented in the actual version of the International System of Cytogenomic Nomenclature (ISCN)^46^.

In this retrospective analysis spanning the past 20 years, we identified several reported systems for identifying and classifying human CHs with numerous variations each. We attribute these discrepancies to the emergence of new techniques, the lack of evaluation standards, and the limited information provided in the scientific literature and databases. We should also add that the size of “normal” heterochromatin blocks on each chromosome has not been established, which also makes its correct assessment difficult. The ideograms displayed in different versions of the ISCN and genome browsers fail to elucidate this issue^28^. Importantly, visualizing CHs properly is difficult, as it is a nonautomated task that depends on experienced cytogeneticists.

To streamline and simplify the scientific discourse, we categorized scoring systems into three primary types distinguished by their methodological approaches, rating levels and assessed chromosome/chromosomal sections (linear measurement, twice the size of the homologous and 16p size comparison model) (Table 4). The Linear Measurement method is the least reported but likely the most accurate, as it assigns exact numerical measurement values. However, extra C-banding is always required and only evaluates the heteromorphisms of 4 chromosomes (1, 9, 16 and Y). The occurrence of different degrees of chromosomal resolution is also a critical point of this methodology. The Twice the Size of the Homologous method is by far the most extensively used and reported system for evaluating CHs. This straightforward methodology can be readily applied in routine G-banding procedures. This technique is effective for all chromosomal resolutions, as comparisons between homologs are conducted within the same metaphase spread. This system assesses all heteromorphic regions in all the chromosomes; however, as heteromorphisms can be present simultaneously in both homologs, this size comparison methodology does not identify and report multiple variants. The 16p Size Comparison model is a simple methodology that can be effectively performed in C-banding or in routine G-banding procedures. This method is efficient for all chromosomal resolutions, as it compares the CHs with the 16p of the same metaphase. However, this comparison model only establishes the upper limit for the size of heterochromatic regions and assesses the heteromorphisms of only 4 chromosomes (1, 9, 16 and Y). To evaluate the hetermorphisms of acrocentric chromosomes, it is necessary to use an additional model (18p SC or TSH for short arms, stalks and satellites). For the centromeric regions, the diameter of a chromatid from the same metaphase is used as a size reference. The combination of the 16p, 18p and chromatid SC models likely represents the most efficient approach to identify and evaluate all the main variable chromosomal sections, as suggested by Liehr in 2016^9^. This publication also suggests minimum size limits to establish the “p-” and “qh-” variants.

**Table 4:** Categorization of scoring systems, applications and limitations.

Scoring System Models	Banding and Methodology	Assessed Regions	Advantages	Limitations
Linear Measurement	CBG image measurement	Pericentromeric 1, 9, 16 and distal Yq	Scoring scale with exact numerical limits	Extra C-banding; not effective for all chromosomal resolutions; only evaluates 4 chromosomes
Twice the Size of the Homologous	GTG/CBG/NOR observation and comparison	All observable heteromorphic regions	GTG evaluation of all chromosomes; effective for all chromosomal resolutions	Failure to identify variants when present in both homologous
16p Size Comparison	GTG/CBG observation and comparison	Pericentromeric 1, 9, 16 and distal Yq	GTG evaluation; effective for all chromosomal resolutions	Only evaluates 4 chromosomes
Trio (16p + 18p + chromatide) Size Comparison	GTG observation and comparison	All observable heteromorphic regions	GTG evaluation of all chromosomes; effective for all chromosomal resolutions	

Currently, there is no chromosome banding assessment system that is effective and comprehensive for evaluating all cytogenetically visible heteromorphic regions. In addition to the well-known heterochromatic blocks present on chromosomes 1, 9, 16, and Y and the acrocentric regions, the centromeric and pericentromeric regions of all chromosomes display variability that must be accurately evaluated. Without an exact description and molecular characterization of all repetitive regions susceptible to heteromorphic changes, such variants can easily be confused with clinically significant gains or losses of chromosomal material^43^. For example, variations in the short arms of acrocentric chromosomes can be due to translocation with Yq12 or with other acrocentric p-arms, amplification or loss of satellite DNA and/or NOR^28,47^. Therefore, only by establishing clear protocols to characterize putative CHs will it be possible to determine which CHs may hint at possible cryptic (unbalanced) translocations, insertions or euchromatic variants, such as chromosome 9 as 9 ph+ and amplification at 9q13^48,49^.

Human heteromorphisms are clearly a controversial topic in genetic diagnosis, and current cytogenetic guidelines do not recommend reporting these variants. The latest version of the ISCN (2020) clarified and reinforced that heteromorphic variants should not be included in the final clinical report. Instead, they should be reserved for the description text that supports the report, where variants may be useful for distinguishing between distinct cell lines or clones^14^. However, this practice is not consensual in clinical cytogenetics or in the scientific community^43^. In a survey conducted by Brothman et al. among cytogeneticists regarding reporting practices for CHs, the responses were not unanimous. Nevertheless, there is a clear consensus on which regions are recognized as heteromorphic and which should be included in reports. The majority of cytogeneticists advocated mentioning pericentric inversions and rare CHs in the final report^46^. In fact, the importance of systematic evaluation and characterization of these variants has been reinforced by several authors, both in the clinical and evolutionary context^2,4,8,12,28,50^. However, the inconsistency in the methodologies and classification systems used to evaluate CHs does not allow accurate reports^15^. For example, the ISCN (2020) suggests a nomenclature for heterochromatic variants of acrocentric short arms that is practically never used in the literature, potentially indicating that this nomenclature is not well clarified or established.

In daily practice, cytogeneticists often rely on parental studies, additional laboratory analyses, and thorough literature reviews to determine whether heteromorphic findings are benign or pathological^46^. This task proves to be problematic, especially in prenatal diagnosis, as there are no defined workflows, and supporting publications are scarce, leading to low-quality reports. Moreover, it is crucial to highlight that relying exclusively on one methodological approach might not offer comprehensive solutions to a particular heteromorphism^28^. Specifically, it is obvious that cytogenetic banding alone is not able to resolve the nature of all CHs; in many cases, fluorescence in situ hybridization is needed to clarify which CH is present in a specific case (see, e.g., CHs of chromosome 9)^16^. The ongoing advancements in computational techniques and new algorithms for identifying repetitive DNA sequences are promising but remain ineffective. The limited length of next-generation sequencing (NGS) reads and significant sequencing error rates in third-generation sequencing (TGS) make it difficult to develop efficient algorithms, imply high detection costs, and provide unsuccessful results^8^. Considering the highly repetitive nature of heterochromatic regions and the challenges associated with sequencing them, molecular methodologies are unlikely to readily replace cytogenetic analyses of CHs^51^. Therefore, reevaluating the importance of establishing clear cytogenetic guidelines and a universal scoring system is imperative.

CHs constitute a natural part of genetic variation; however, they can exert significant effects on gene and phenotype expression. Understanding CHs is crucial for gaining insights into genome evolution, gene regulation and disease mechanisms. Nevertheless, CHs remain a challenge in genetic diagnostics and are often not adequately assessed or reported in routine cytogenetic analysis. The present study provides a comprehensive understanding of the diverse methodologies and classification systems for heterochromatic CHs outlined in the literature and exposes the inconsistencies and difficulties in correctly evaluating and reporting these variants. Currently, the literature and databases still lack descriptions of the standard sizes attributed to heterochromatic regions and the exact characterization and documentation of numerous human CHs. The absence of a universal scoring system hinders the accurate identification of CHs, preventing the scientific community from assigning a precise value to their impact on genetic diagnosis. This lack of standardization also compromises scientific reproducibility, potentially leading to erroneous conclusions. Therefore, it is crucial to establish robust standardization in the evaluation of human CHs and carefully consider the advantages and disadvantages of reporting these variants. Such efforts can be achieved only through cooperation and collaboration within the global cytogenetic scientific community. Furthermore, it is crucial to highlight that the use of multiple techniques to address the same issue often uncovers the harmless or pathological essence of the variation. Ultimately, we emphasize the importance of strengthening global scientific research on human heterochromatic regions and their variations, as well as advanced detection technologies, to unravel their intricate structures, functions, interactions and clinical implications.
